# Overexpression of the *mTERT* gene by adenoviral vectors promotes the proliferation of neuronal stem cells *in vitro* and stimulates neurogenesis in the hippocampus of mice

**DOI:** 10.7555/JBR.26.20110078

**Published:** 2012-04-18

**Authors:** Mengying Liu, Yao Hu, Lijuan Zhu, Chen Chen, Yu Zhang, Weixiang Sun, Qigang Zhou

**Affiliations:** Department of Pharmacology, Pharmacy College, Nanjing Medical University, Nanjing, Jiangsu 210029, China.

**Keywords:** telomerase, construct, eukaryotic expression vector, adenoviral vector, proliferation, neuronal stem cells

## Abstract

We sought to construct the adenoviral vector carrying the gene encoding mouse telomerase reverse transcriptase (mTERT), as well as detect its expression and effect on the proliferation of neuronal stem cells. *mTERT* was amplified by RT-PCR and then the eukaryotic expression vector of pDC-EGFP-TERT was constructed. After DNA sequence analysis, we detected that there were 293 cells transfected with pDC-EGFP-TERT and helper adenovirus plasmid pBHG lox ΔE1, and three Cre using Lipofectamine 2000 mediation, named Ad-mTERT-GFP, to package adenoviral particles. The Ad-mTERT-GFP was used to infect neuronal stem cells and then the expression and activity of mTERT were detected. In addition, Bromodeoxyuridine labeling test identified the impact of mTERT overexpression on proliferation of neuronal stem cells. The recombinant adenoviral vector confirmed that mTERT was successfully constructed. Overexpression of mTERT stimulated the proliferation of neuronal stem cells both *in vitro* and *in vivo*. mTERT overexpression *via* adenoviral vector carrying *mTERT* cDNA upregulated the ability of proliferation in neuronal stem cells.

## INTRODUCTION

Telomerase is a complex consisting of a protein, a telomerase reverse transcriptase (TERT), and an RNA component known as telomerase RNA (TERC). TERT is used as a template for the addition of short, G-rich repeat at the ends of chromosomes[1]–[3]. Extensive studies reveal that telomerase influences several processes of the cell life cycle including proliferation, differentiation, survival, and death[4]–[6]. Particularly, telomerase is present, at very high levels, in neural precursor cells including adult neuronal stem cells in the dentate gyrus (DG) of the hippocampus, in which they disappear after their maturation into neurons[6],[7].

Telomerase has been considered to be closely related to the proliferation of stem cells such as tumor cells and neuronal stem cells. It may also play an essential role in oncogenesis, aging, and neurodegeneration[8],[9]. Previous studies have shown that *TERT* knockout mice display a marked decline in neuronal stem cell proliferation and a selective impairment in neuronal differentiation and survival[10]. Moreover, TERT promotes cardiac muscle cell proliferation, hypertrophy, survival[11], and the proliferation of hair follicle stem cells[12]. Our previous study also confirmed that 3′-azido-deoxythymidine (AZT), a telomerase inhibitor, disrupted neurogenesis *in vivo* and *in vitro*[13]. Although the previous study established a potential link between telomerase and the growth of neuronal stem cells, this theory lacks critical evidence that telomerase overexpression could promote neuronal stem cell growth. It is still unknown whether overexpression of TERT affects the proliferation of neuronal stem cells. To address this question, we constructed adenovirus vectors carrying the entire gene encoding mouse TERT. Importantly, this recombinant adenovirus vector will be useful in investigating and understanding the function of telomerase in neurogenesis, tumor growth and cell injury. It is concluded that our research could lead to novel approaches to preventing cancer, aging, and neurodegenerative diseases.

## MATERIALS AND METHODS

### Reagents

DMEM/F12, neurobasal media and B27 were purchased from Gibco (Carlsbad, CA, USA). TRIzol, 2×PCR MasterMix, and Lipofectamine 2000 were purchased from Invitrogen (Carlsbad, CA, USA). T4 DNA ligase and *Eco*RI, *Bam*HI and *Age*I were purchased from NEB Company (Carlsbad, CA, USA). pGC-FU vector was purchased from Genechem Company (Shanghai, China). All the antibodies were purchased from Santa Cruz Biotechnology (Santa Cruz, CA, USA) or Chemicon (Billerica, MA, USA). The pDC315-GFP vectors were purchased from Shanghai Genechem Company Ltd., Shanghai, China.

### Cell culture

As described previously[14], embryonic neuronal stem cells were isolated from the mouse cortex and hippocampus at embryonic d 14 (E14). Cells were grown as suspension in medium containing 20 ng/mL basic fibroblast growth factor (bFGF, Sigma-Aldrich, St. Louis, MO, USA), 20 ng/mL epidermal growth factor (EGF, Sigma-Aldrich), and 2% B27 supplement (Invitrogen, Grand Island, NY, USA), and were passaged every 4-6 d. These embryonic neuronal stem cells had the capacity for proliferation and self-renewal, and were able to generate differentiated progenies until the 10^th^ passage. Embryonic neuronal stem cells of the 2^nd^ to 10^th^ passage were used in this study.

For bromodeoxyuridine (BrdU) incorporation and co-culture experiments, embryonic neuronal stem cells were planted on coverslips (2 cm×2 cm) coated with polyornithine (10 µg/mL; Sigma-Aldrich) and laminin (5 µg/mL; Invitrogen) at 1×104 cells/cm^2^, cultured as a monolayer.

### Telomerase activity assay

Telomerase activity of cultured neuronal stem cells was detected using TRAPEZE XL telomerase detection kit (Millipore, Billerica, MA, USA). Single-cell suspension of neuronal stem cells was seeded in uncoated dishes, and cultured for 96 h to form moderate neurospheres. The neurospheres were then collected and lysed with CHAPS lysis buffer. Telomerase activity was detected with the telomeric repeat amplification protocol (TRAP)[14] with some modifications. Fluorescence energy transfer primers were used to generate fluorescently labeled TRAP products, which were quantitatively measured with fluorescence plate reader (SpectraMax M2e, Molecular Devices, Sunnyvale, CA, USA) or visualized following polyacrylamide gel electrophoresis on a 10% nondenaturing gel and SYBR Green I (Invitrogen) staining.

### Adenovirus generation, production, and purification

The coding sequence of *mTERT* was amplified by RT-PCR. The PCR fragments and the pDC315-GFP plasmid were digested with *Eco*R I and then ligated with T4 DNA ligase to construct plasmid pDC315-mTERT-GFP. The plasmid was transformed to competent DH5α *Escherichia coli* for identification. The primer TERE-SEQF was located in the target gene and the primer EGFP-C-R was located in the vector; they were designed to ensure the success of directional linkage. The primer (TERE-SEQF and EGFP-C-R) sequences were as follows: forward, 5′-TGAACAGCCTCCAGACAGTC-3′; reverse, 5′-CGTCGCCGTCCAGCTCGACCAG-3′. For recovery of recombinant adenoviruses (Ad-mTERT-GFP), the pDC315-mTERT-GFP plasmid, which encodes mTERT-GFP, was used to co-transfect HEK293 cells with plasmid pBHG lox ΔE1,3 Cre. Transfection solutions were prepared by mixing 5 µg of the pDC315-mTERT-GFP plasmid, 5 µg of the pBHG lox ΔE1,3 Cre plasmid and 10 µL Lipofectamine 2000 in 50 µL antibiotics-free DMEM. After 8 d, the supernatant was harvested from HEK 293 cells. After 3 rounds of virus amplification, the supernatant was filtered at 0.45 µm, and purified using Adeno-X™ virus purification kit (Clontech Laboratories, Mountain View, CA, USA). After resuspension, serially diluted adenovirus was used to transduce HEK293 cells. After 7 d, labeled HEK293 cells were counted to calculate the viral titer (≤2.5×10^10^ pfu/mL). A fluorescence microscope could observe the effects of transfection *in vivo.*

### Western blot analysis

As described previously[15], the cells were homogenized in ice-cold lysis buffer containing 100 mmol/L HEPES, 200 mmol/L NaCl, 10% glycerol, 2 mmol/L Na4P2O7, 2 mmol/L dithiothreitol, 1 mmol/L EDTA, 1 mmol/L benzamidine, 0.1 mmol/L Na3VO4, 1 µmol/L pepstatine, 10 µg/mL aprotinin, 10 µg/mL leupeptin, and 10 µmol/L phenylmethylsulfonyl fluoride (pH 7.4). After lysis for 15 min, the samples were centrifuged at 20,000 *g* for 15 min. The samples containing equivalent amounts of protein (20 µg) were subjected to 8% sodium dodecyl sulfate-polyacrylamide gel electrophoresis. The separated proteins were transferred onto nitrocellulose membranes (Millipore Co., Billerica, MA, USA) overnight at 4°C. Blotting membranes were incubated with blocking solution [5% non-fat dried milk powder dissolved in Tris-Buffered Saline Tween-20 (TBST) buffer (pH 7.5, 10 mmol/L Tris-HCl, 150 mmol/L NaCl, and 0.1% Tween 20)] for 1 h at 22-25°C, washed three times, and incubated with mouse anti-mTERT (1:1,000, Santa Cruz Biotechnology), or rabbit anti-GFP (1:2,000, Chemicon) antibodies, in TBST overnight at 4°C. Internal control was carried out using GAPDH. After several washes with TBST, the membranes were incubated for 1 h with horseradish peroxidase-conjugated secondary antibody. The membranes were then processed with enhanced chemiluminescence Western blotting detection reagents (Pierce, Rockford, IL, USA). After that the films were scanned, and densitometry was performed by Quantity One Image Software (Bio-Rad, Irvine, CA, USA). The relative level of the protein was quantified from the scanned films.

### RT-PCR

Total RNA was extracted using Trizol reagent according to the manufacturer's instructions (Invitrogen). The primer sequences were listed as follows: for *mTERT*, forward, 5′-GTAGAACGCAGATCGAATTCATGACCCGCGCTCCTCG-3′; reverse, 5′-CCCTTGCTCACCATGAATTCGTCCAAAATGGTCTGAAAGTC-3′; for identification of positive clones, forward 5′-TGAACAGCCTCCAGACAGTC-3′; reverse, 5′- CGTCGCCGTCCAGCTCGACCAG-3′. The PCR was run for 30 cycles at 94°C for 30 s, 55°C for 30 s, and 72°C for 3 min. The PCR products were separated by electrophoresis through a 1.5% agarose gel containing 0.5% µg/mL ethidium bromide and imaged using a BioDoc-IT imaging system (Bio-Rad).

**Fig. 1:**
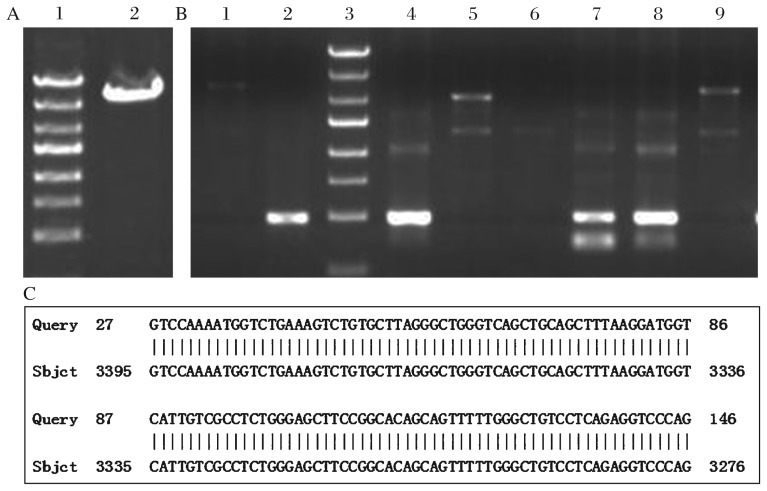
Construction of the expression vector pDC315-EGFP-mTERT. A: mTERT cDNA (3,426bp). lane 1:DNA marker, lane 2: sample. B: Recombine plasmid was transformed into competent DH5α *Escherichia coli* strains. The positive clones were randomly coded and we chose No. 1-6# clones to extract plasmid DNA for PCR amplification. The primers which were named as TERE-SEQF and located in the target gene and the primer EGFP-C-R, which was located in the vector, were designed to ensure the success of directional linkage. Lane 1: negative control; lane 2: positive control; lane 3: DNA marker; lane 4-9: the PCR production of 1-6# clones. The 426 bp long products (pDC315-EGFP-mTERE) were yielded in No. 1# (lane 5), 4# (lane 7), 5# (lane 8) clones and positive control (lane 2), indicating that the cDNA of mTERT was successfully cloned into pDC315-EGFP plasmid. C: a representative part of the gene order of the target gene in the vector and the comparative analysis to the *mTERT* gene.

### Animal protocol

Pregnant ICR mice were purchased from Nanjing Medical University Experiment Animal Center. All procedures involving the use of animals were approved by the Institutional Animal Care and Use Committee of Nanjing Medical University. C57BL/6 mice weighing 18-22 g were used for the experimental procedures. Mice were anesthetized with a solution of chloral hydrate (30 mg/kg, intraperitoneal). A volume of 2 µL Ad-mTERT-GFP or Ad-GFP at the titer of 2.5×10^10^ pfu/mL was infused into the DG (0.2 µL/min) at coordinates: 2.3 mm posterior to bregma, 1.3 mm lateral to the midline, and 2.0 mm below dura. Mice were allowed 1 week to acclimate before experimentation. Animals were housed in plastic cages with bedding. Food and tap water were available *ad libitum* for the duration of the experiments unless otherwise noted. The temperature was maintained at 22±2°C. A 12 h light/dark cycle was maintained, with lights on at 6:00 am.

### Statistical analysis

All statistical analyses were performed using the statistical software Statistical Package for the SPSS 11.0 (SPSS Inc., Chicago, IL, USA). Comparisons between the two groups were made with the two-tailed Student's *t*-test. All data were presented as mean±SEM. Statistical significance was accepted at *P* < 0.05.

## RESULTS

### Construction of the expression vector pDC315-EGFP-mTERT

mTERT cDNA was cloned from the embryonic hippocampus and agarose gel electrophoresis of the PCR products yielded an expected band, 3,426 bp in size (***Fig. 1A***). The PCR products were inserted into pDC315-EGFP. PCR analysis of the cloned plasmids followed by gene sequencing showed that the cDNA of mTERT was successfully and correctly cloned into pDC315-EGFP plasmid (***Fig. 1B***) and the cloned gene was the same as *mTERT* (GENE ID: 21752 mTERT) in the GenBank (***Fig. 1C***). These findings indicated that the expression vector pDC315-EGFP-mTERT was successfully constructed.

### Adenovirus vectors packaging, purification and identification

**Fig. 2:**
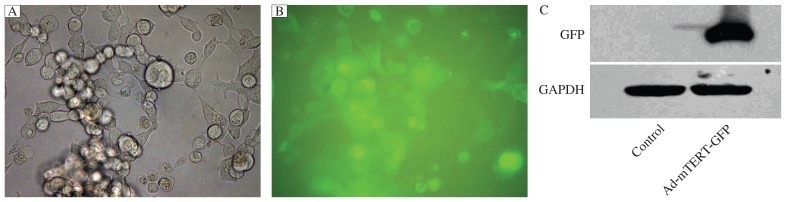
Detection of HEK 293 cells after infection with adenovirus vectors. Representative photos (A, B) of HEK 293 cells infected with adenovirus vectors. C: immunoblots showing GFP levels in HEK 293 cells infected with or without Ad-mTERT-GFP.

We generated recombinant adenovirus, Ad-mTERT-GFP, expressing mTERT fused with GFP. After several cycles of amplification, the final virus titers were about 2.5×10^10^ pfu/mL. We infected HEK 293 cells with these adenoviral vectors and found that, at d 7 post infection, 90% of the cells were GFP positive, indicating success of adenovirus vector packaging (***Fig. 2A***). Consistently, a high level of GFP was detected by Western blotting assays in HEK 293 cells infected with the packaged adenovirus, but not in the control HEK 293 cells (***Fig. 2B***).

**Fig. 3:**
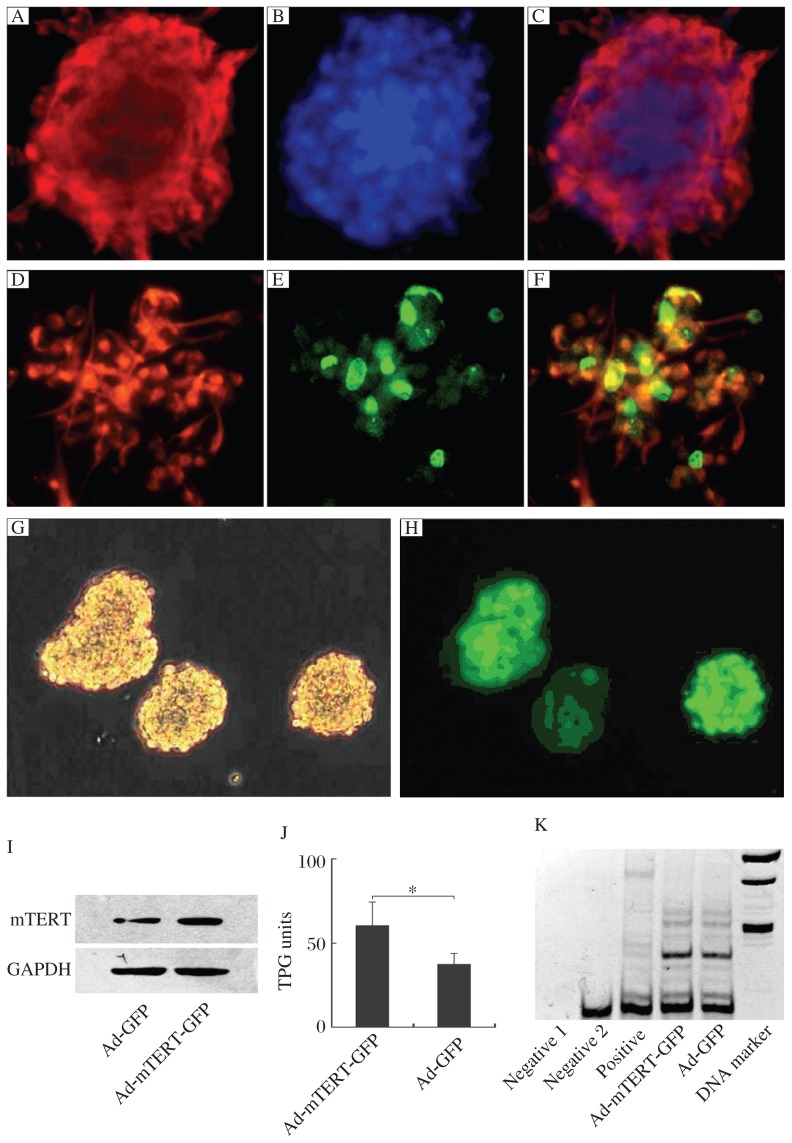
Detection of neuronal stem cells (NSCs) after infection with adenoviral vectors. Identification of cultured NSCs. Fluorescent images of neurospheres stained by nestin (A) and Hochest (B), which were merged in C. Fluorescent images of monolayer-cultured NSCs stained by nestin (D) and BrdU (E), which were merged in F. Representative photos of NSCs infected with adenovirus vectors under light (G) or fluorescence microscopy (H). I: Immunoblots showing mTERT levels in NSCs infected with Ad-mTERT-GFP or with Ad-GFP. J: Telomerase activity of NSCs infected with Ad-mTERT-GFP was much higher than that of NSCs infected with Ad-GFP (60.34±13.87 *vs* 37.19±6.74, *P* < 0.05). K: The representative of SYBR Green I-stained gel visualizing TRAP products. Negative 1: a *Taq* enzyme-free control; Negative 2: a cell-free control; Positive: a cell line expressing high level of telomerase. TPG: total product generated; TRAP: telomeric repeat amplification protocol.

### Infection of neuronal stem cells with adenoviral vectors

We cultured neuronal stem cells which were positive for nestin, a marker of stem cells, as shown by immunohistochemistry (***Fig. 3A*** to ***3F***); almost all the cells in the neurospheres were labeled with nestin. Additionally, BrdU incorporation studies showed that 2 h after BrdU (10 µmol/L) incubation, approximately 35% of the neuronal stem cells were labeled with BrdU, indicating that the cultured neuronal stem cells were dividing (***Fig. 3A*** to ***3F***). For determination of the efficiency of infection, we infected cultured neuronal stem cells with Ad-mTERT-GFP at a titer of 2.5×10^7^ pfu/mL. Approximately 90% of the cells expressed GFP 2 d after infection (***Fig. 3G*** and ****3H****). Western blot analysis, by using antibody against mTERT, demonstrated that mTERT was overexpressed in cells infected with Ad-mTERT-GFP when compared with those cells infected with Ad-GFP (***Fig. 3I***). In addition, TRAP assays showed that neuronal stem cells infected with Ad-mTERT-GFP possessed markedly higher telomerase activities, compared with those infected with Ad-GFP (***Fig. 3J*** and ****3K****).

### mTERT overexpression stimulated the proliferation of neuronal stem cells

**Fig. 4:**
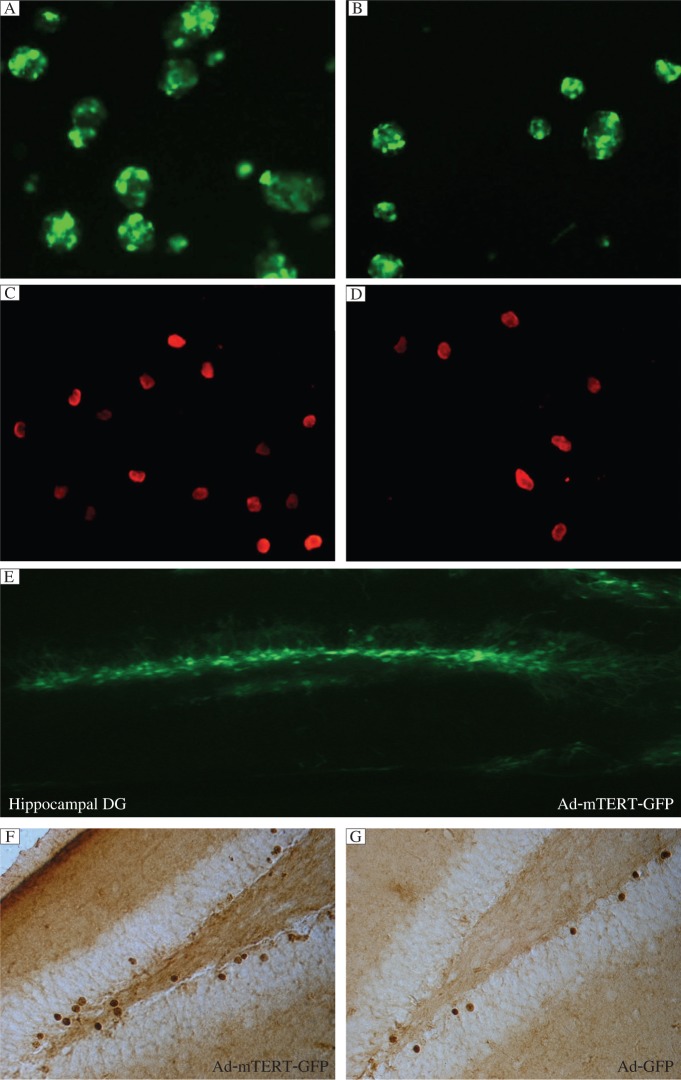
Overexpression of mTERT stimulates proliferation of neuronal stem cells (NSCs). A: Representatives of newly formed embryonic neurospheres 24 h after infection with Ad-mTERT-GFP (A) or Ad-GFP (B). Representatives of Brdu-positive cells after 22 h after infection with Ad-mTERT-GFP (C) or Ad-GFP (D). E: Representative DG area with Ad-mTERT-GFP transfection. Representative BrdU-positive cells in the dentate gyrus (DG) of mice transfected with Ad-mTERT-GFP (F) or Ad-GFP (G) at 28 d after BrdU labeling. Data were normalized to the percentage of control.

To investigate the effect of mTERT overexpression on proliferation of neuronal stem cells, we infected cultured neuronal stem cells with Ad-mTERT-GFP or Ad-GFP. Twenty-four h later, we observed that all neurospheres showed green fluorescence and the number of cells in Ad-mTERT-GFP wells was more than that in Ad-GFP wells (98±5.4 /cm^2^ *vs* 69.3±3.12 /cm^2^, *P* < 0.05, *n* = 4). In addition, the mean size of neurospheres in Ad-mTERT-GFP wells was larger than that of Ad-GFP wells (data not shown). We found that mTERT overexpression increased the proliferative potency of neuronal stem cells *in vitro* [(124±12.78)×10^5^ cells/mL for Ad-mTERT-GFP wells, and (71.25±4.04)×10^5^ cells/mL for Ad-GFP wells, *P* < 0.05, *n* = 4] (***Fig. 4A*** and ****4B****). In addition, 10 µmol/L BrdU was added to label the dividing cells 22 h after infection. Similar results were obtained with BrdU incorporation experiments using monolayer-cultured neuronal stem cells (***Fig. 4C*** and ****4D***, P* < 0.05). However, it remains unknown whether *mTERT* overexpression in adult hippocampal DG upregulates neurogenesis. To prove this hypothesis, we delivered 2 µL Ad-mTERT-GFP or Ad-GFP at the titer of 2.5×10^10^ pfu/mL into the bilateral hippocampus of mice by stereotaxic microinjection. These mice were then treated with BrdU at 48 h after microinjection. Numerous cells with GFP fluorescence were observed in the DG 5 d after transfection (***Fig. 4E***) and the mRNA level of mTERT was obviously increased (*vs* Ad-GFP, data not shown), indicating the success of overexpression of mTERT in the DG. At d 28 after transfection, the hippocampus transfected with Ad-mTERT-GFP (***Fig. 4F***) exhibited markedly increased BrdU positive cells in the DG, compared with the Ad-GFP group (***Fig. 4G***) (*P* < 0.01, *n* = 5), suggesting an increase in neurogenesis in the adult period by mTERT overexpression.

## DISCUSSION

High levels of telomerase activity have been associated with cancer by causing increased proliferation of tumor cells[8],[9]. In this study, we constructed adenovirus vector carrying the whole cDNA of mTERT and showed that overexpression of the catalytic subunit of telomerase stimulates the proliferation of neuronal stem cells. This finding indicates that high levels of telomerase may be involved in physiological functions of organs and the pathogenesis of disorders in the central neuronal system.

Telomerase allows immortal growth of stem cells by maintaining the telomere at a constant length as cells divide. High levels of telomerase activity are associated with cell proliferation during embryonic development and transformation of cells, and the development of cancers[8],[9]. However, whether artificial overexpression of telomerase could contribute to the induction of division of neuronal stem cells remains unknown. Telomerase activity is dependent on TERT, a specialized type of reverse transcriptase[16]–[17]. Accordingly, we used adeno-associated virus to deliver the whole cDNA of mouse TERT into cultured neuronal stem cells. The evidence demonstrated the overexpression of mTERT in the neuronal stem cells by adenovirus vectors was successful. The data from this study suggests that telomerase may be an essential regulator of neuronal stem cell proliferation. Consistently, previous studies revealed that several factors upregulate neurogenesis, including brain-derived neurotrophic factor (BDNF), cAMP response element-binding protein (CREB), *Homo sapiens* v-myc myelocytomatosis viral oncogene homolog (C-MYC), activate transcription, and expression of TERT[6],[14],[18]. Inhibition of telomerase repressed the proliferation of stem cells, including neuronal stem cells both *in vivo* and *in vitro*[13],[19]. In addition, nNOS in the cytoplasm from neurons and in the cytoblast from neuronal stem cells regulate neurogenesis via bidirectional modulation of telomerase activity[14]. These pieces of evidence, altogether, imply that telomerase may be the primary factor in the final common pathway affecting the self-renewal and dividing capability of stem cells.

Although disappearing in somatic cells after differentiation, telomerase is normally expressed in stem cells and in germ-cell lineages in adulthood[20]. Especially in the adult brain, TERT expression is observed in the places primarily limited to where neurogenesis occurs daily, including the hippocampus, the lateral cerebral ventricle and the olfactory bulbs[21],[22]. It is believed that the perturbations in adult hippocampal neurogenesis are relevant to diverse physiological and pathological processes such as learning and memory, epilepsy, schizophrenia, depression, and stroke[8],[23]–[27]. More importantly, reduced hippocampal neurogenesis has been demonstrated as the cause of deficits in learning and memory function[28], and hippocampal neurogenesis is required for the antidepressive effects of antidepressants[29]. As it is hopeful that specific gene-expressing adeno-vectors can be designed to treat diseases, our work provides initial evidence and potential intervention strategy for curing these disorders.
